# A model-guided method for improving coronary artery tree extractions from CCTA images

**DOI:** 10.1007/s11548-018-1891-7

**Published:** 2018-11-28

**Authors:** Qing Cao, Alexander Broersen, Pieter H. Kitslaar, Boudewijn P. F. Lelieveldt, Jouke Dijkstra

**Affiliations:** 1 Division of Image Processing, Department of Radiology, Leiden University Medical Center, Leiden, The Netherlands; 2 Medis Medical Imaging Systems BV, Leiden, The Netherlands

**Keywords:** Coronary computed tomography angiography, Coronary artery tree, Coronary artery anatomy, Quality score

## Abstract

**Purpose:**

Automatically extracted coronary artery trees (CATs) from coronary computed tomography angiography images could contain incorrect extractions which require manual corrections before they can be used in clinical practice. A model-guided method for improving the extracted CAT is described to automatically detect potential incorrect extractions and improve them.

**Methods:**

The proposed method is a coarse-to-fine approach. A coarse improvement is first applied on all vessels in the extracted CAT, and then a fine improvement is applied only on vessels with higher clinical significance. Based upon a decision tree, the proposed method automatically and iteratively performs improvement operations for the entire extracted CAT until it meets the stop criteria. The improvement in the extraction quality obtained by the proposed method is measured using a scoring system. 18 datasets were used to determine optimal values for the parameters involved in the model-guided method and 122 datasets were used for evaluation.

**Results:**

Compared to the initial automatic extractions, the proposed method improves the CATs for 122 datasets from an average quality score of 87 ± 6 to 93 ± 4. The developed method is able to run within 2 min on a typical workstation. The difference in extraction quality after automatic improvement is negatively correlated with the initial extraction quality (*R *= − 0.694, *P* < 0.001).

**Conclusion:**

Without deteriorating the initially extracted CATs, the presented method automatically detects incorrect extractions and improves the CATs to an average quality score of 93 guided by anatomical statistical models.

**Electronic supplementary material:**

The online version of this article (10.1007/s11548-018-1891-7) contains supplementary material, which is available to authorized users.

## Introduction

Coronary computed tomography angiography (CCTA) is an established technique for the assessment of patients with suspected coronary artery disease (CAD) [1]. In order to diagnose CAD on CCTA images, the coronary artery tree (CAT) is often extracted. Automatic CAT extraction methods based on minimum path techniques have been widely used due to their simplicity and computational efficiency [2–4]. The minimum path searching is often performed on a vesselness image which is typically created by applying a modified Frangi’s vesselness filter to CCTA images [5, 6].

However, severe occlusion or low contrast in coronary arteries can result in gaps in the vesselness image. Furthermore, surrounding veins could be wrongly extracted as arteries because of their similar appearance. These situations can create undesirable shorter or longer extractions which require manual corrections from experts. Han et al.  [7] proposed active searching to solve the discontinuity in automatically extracted CATs using a statistical branch occurrence location model which predicts the position of a branch. However, their model is used only for the left main artery and discontinuity detection was not described. Zheng et al. [8] used a 3D coronary tree model to predict the initial position of the major centerlines while side branch information is not included.

This paper presents a model-guided method for improving the extracted CAT from CCTA images. Guided by anatomical statistical models, the proposed method automatically detects potential incorrect extractions and improves the extracted CAT. A recently proposed scoring system by Cao et al. [9] is exploited to monitor the quality of the CAT improved by the proposed method.

## Methods

**Fig. 1:**
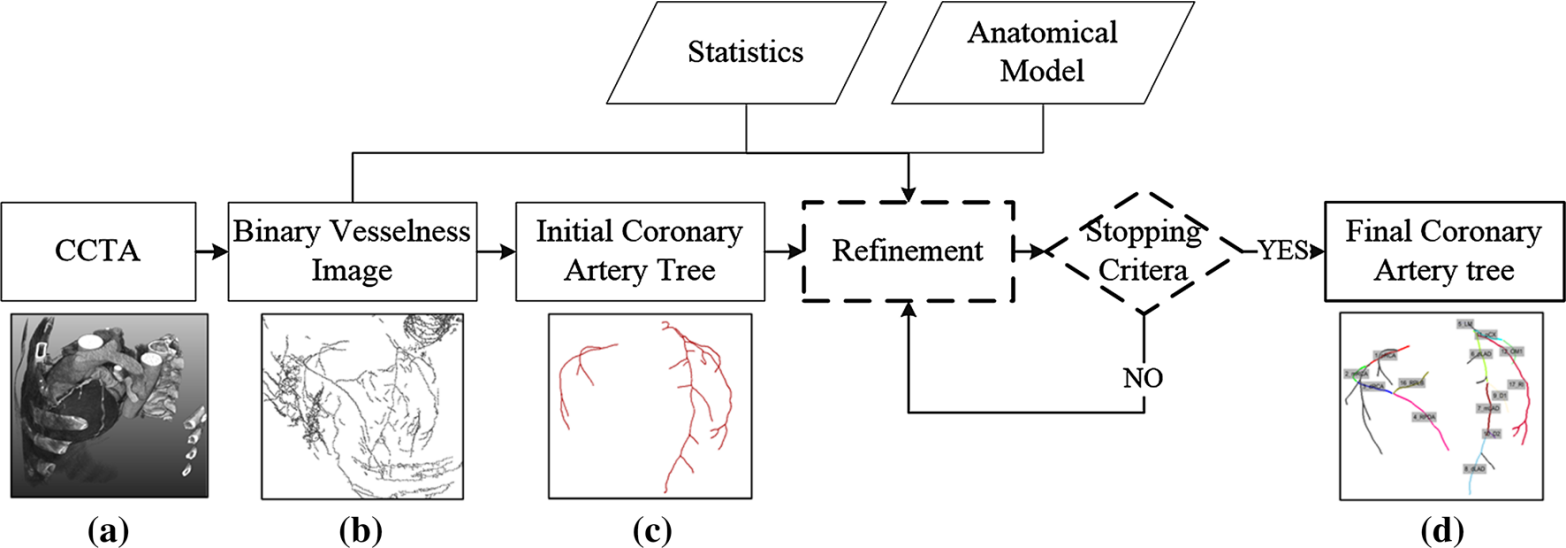
Pipeline of the model-guided method for improving coronary artery tree extractions. Modules in dashed boxes are described in more detail in this paper. **a** Coronary computed tomography angiography image. **b** Binary vesselness image. **c** Fully automatically extracted coronary artery tree. **d** Automatically improved extraction using the proposed model-guided method

Figure 1 gives an overview of the proposed method. On the CCTA image (a), an improved Frangi’s vesselness filter is applied to create a binary vesselness image (b). Next, an initial CAT (c) is extracted using a fully automatic extraction method presented by Yang et al. [6]. Then, the proposed method based upon the anatomical statistical model and binary vesselness image is applied to the initial CAT. The improvement process is iteratively performed by adding missing arteries and removing wrong extractions until the stop criteria are met. The automatically improved CAT together with the anatomical names assigned to the corresponding coronary artery segments is shown in (d). Details of the model-guided improvement method are described in the following sections. We first introduce some prior knowledge in the next section.

### Coronary artery tree

**Fig. 2:**
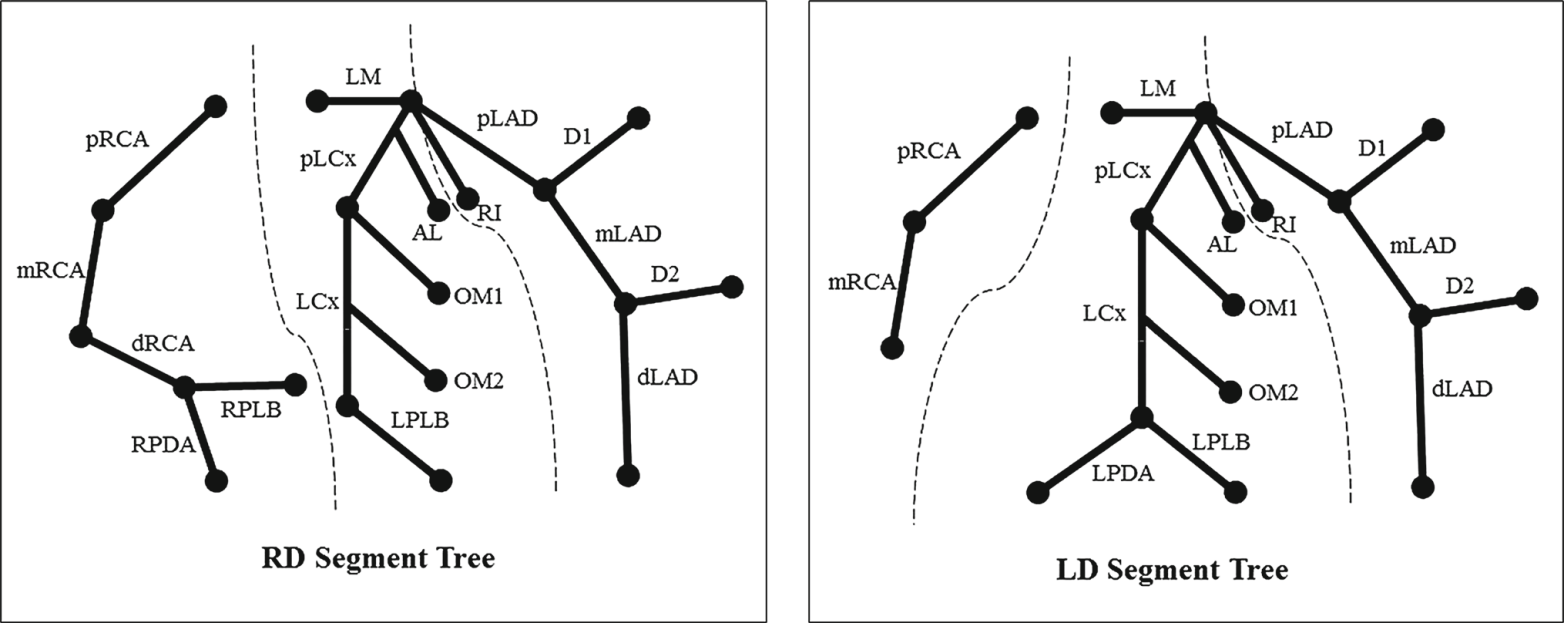
RD and LD coronary artery tree segments. Dashed lines represent division between sub-trees RCA, LAD, and LCX. *RCA* right coronary artery, *LAD* left anterior descending, *LCX* left circumflex, *LD* left dominant, *RD* right dominant

A CAT is composed of three main branches, right coronary artery (RCA), left anterior descending (LAD) and left circumflex (LCX), and their side branches. The main branch that supplies the posterior descending artery (PDA) determines the CAT dominance type [10]: right dominant (RD), left dominant (LD), and balanced type. Different dominance types have different geometrical topologies. To report CAD on CCTA images, the modified 17-segments model defined by the American Heart Association (AHA) is widely used in clinical practice [11, 12]. Figure 2 shows the AHA models for RD and LD cases as two separate schematic figures. The balanced type will be treated as RD which is the most prevalent dominance type. Main branches are divided into proximal (p-), mid (m-), and distal (d-) segments and these segments are represented as parent–child relationships.

*The initial extractions* The initial CAT we used in this presented work is extracted using the automatic centerline extraction method proposed by Yang et al. [6]. Additionally, a binary vesselness image containing the central axes of the vessel-like structures of the CCTA image is created during the extraction process (Fig. 1b).

*The statistical models* Cao et al. [9] defined separate anatomical statistical models for RD and LD cases which contain global information for all vessels in a CAT as well as local information for each specific segment in the AHA model. In the statistical models, each label has a weight to represent its importance. An important label has a higher weight and weight values for each label can be found in “Supplementary Material Section 1.” The deviations of the extracted CAT from the anatomical statistical models indicate the locations of the incorrect extractions.

### The model-guided method

Guided by the statistical models mentioned above, the initial CAT extractions are improved using a coarse-to-fine approach, starting with the coarse improvement on all vessels in the extracted CAT followed by the fine improvement only on the segments with corresponding anatomical definitions in the AHA model which are further referred as *vessels*-*with*-*labels.*

### Coarse improvement

The coarse improvement removes incorrectly extracted segments in three steps. First, vessel-like structures in the initial CAT not connected with the left and right ostia are removed. Second, pathlines longer than the corresponding global maximum length from the statistical model are pruned to the maximum length. Pathlines shorter than 1 mm are considered as not important and removed. Third, vessels in the initial CAT with angles larger than expected from the statistical model are removed. The angle between the parent branch along the blood flow direction and the side branch direction is calculated and the vessel is removed when the angle is larger than 120°. Examples of applying coarse improvement on CATs can be found in “Supplementary Materials Section 2.”

### Fine improvement

Coarse-improvement operations are mainly shortening and removing while the fine improvement performs a more precise analysis on *vessels*-*with*-*labels* to verify correctness of the anatomical positions and to extract missing arteries in a CAT.

*Automatic detection of inaccurate extractions* On the coarsely improved CAT, anatomical labels are automatically assigned using the labeling method of Cao et al. [13]. By comparing all anatomical labels in the model with *vessels*-*with*-*labels* in the extracted CAT, their presence and absence in the extracted CAT are obtained. If an anatomical label is present in the CAT but the length of the segment is outside the anatomical statistical model range for this specific label, it will be marked as too short or too long. If an anatomical label is absent, it could be a missed extraction or a normal variation since some labels appear more often than the others. We use the label weight to decide the probability of a miss-extraction when the label is absent. To improve the above identified inaccurate extractions, two improvement operations, deletion and extension, are performed.

#### Deletion

**Fig. 3:**
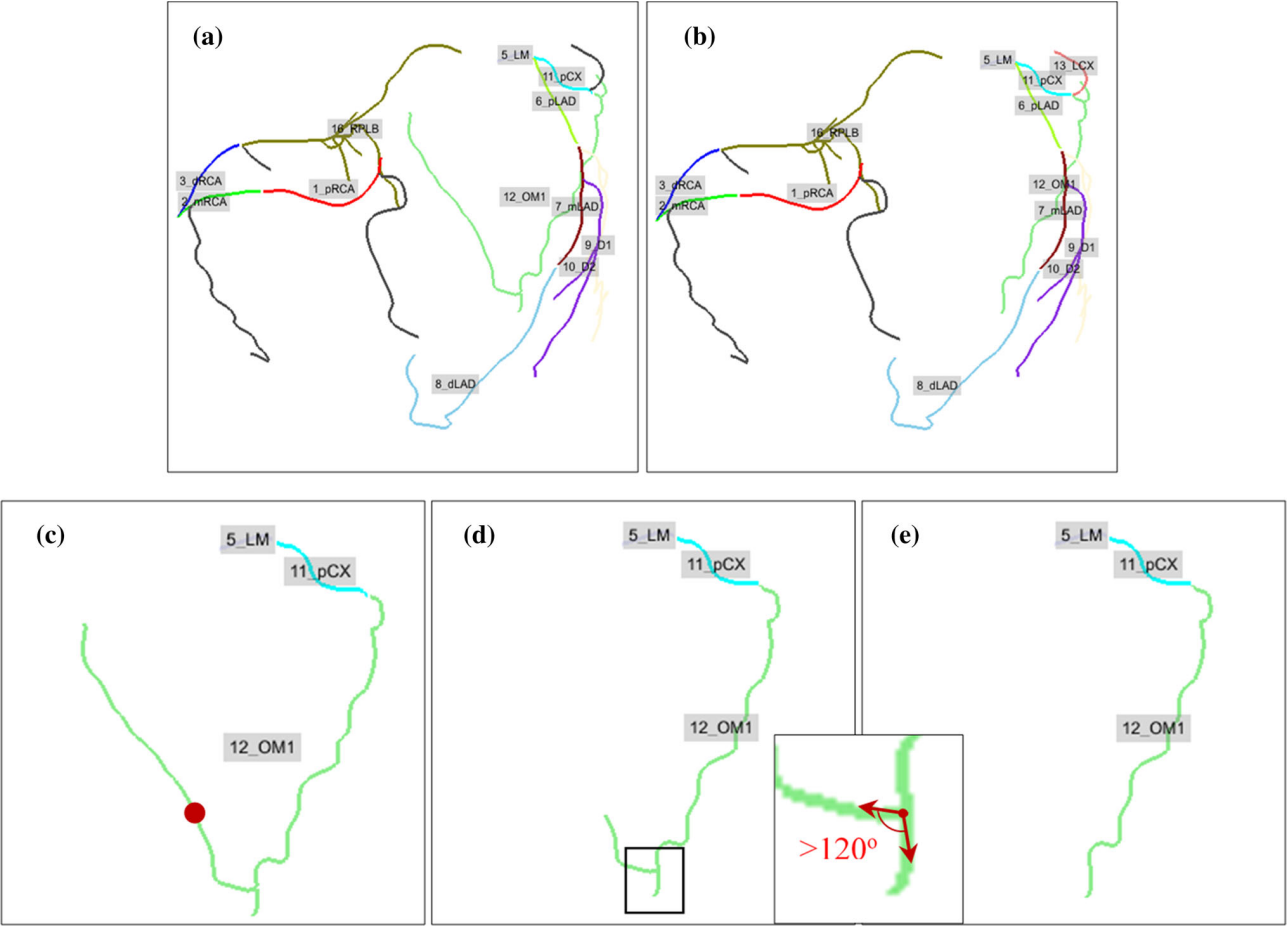
Automatic long vessel deletion and angle improvement. **a** Initially extracted CAT with labels. **b** Automatically improved CAT; **c**–**e** zoom-in image of the OM1. **c** Red point on OM1 shows the position of the maximum length of the OM1. **d** Shortened OM1 with side branch bifurcation angle larger than 120°. **e** OM1 after length and angle improvement. *CAT* coronary artery tree, *OM1* first obtuse marginal branch

If a labeled segment is too long, a deletion operation similar to the one described in the coarse improvement section is performed. The segment is deleted from where it starts exceeding the maximum length. Since other improvements can alter the topology of a CAT, the vessel angles are computed again and labeled segments with angles larger than 120° are deleted. Figure 3 shows an extracted CAT which is improved by the deletion operation.

#### Extension

If a labeled segment is too short or an important label is absent, an extension operation is performed to bridge the gaps in the binary vesselness image. Yang et al. [6] presented branch searching on the binary vesselness image using a wave propagation algorithm with a fixed searching distance. Their method considers all points in the extracted CAT with a large change in curvature as starting points. Since the inclusion of all points is computing-intensive and a fixed search distance cannot deal with gaps of different sizes, we introduce an improved branch searching algorithm using automatically determined start points and an adaptively-set searching distance.

*Improved branch searching* The improved branch searching method selects the start points depending on the situation. (1) If a side branch label is absent, all points on the parent label with a large curvature are selected as start points. (2) If a main branch label, such as mRCA, is absent, all points on the extracted main branch with large curvature are used as start points. This is because the identification of the whole main branch could be incorrect if a main branch segment is absent as mentioned in the automatic identification method [13]. An example is presented in “Supplementary Material Section 3.1” to illustrate the difference in selecting starting points to search for a main branch label. (3) If a labeled segment is too short, the end point of this segment is selected as the start point.

**Fig. 4:**
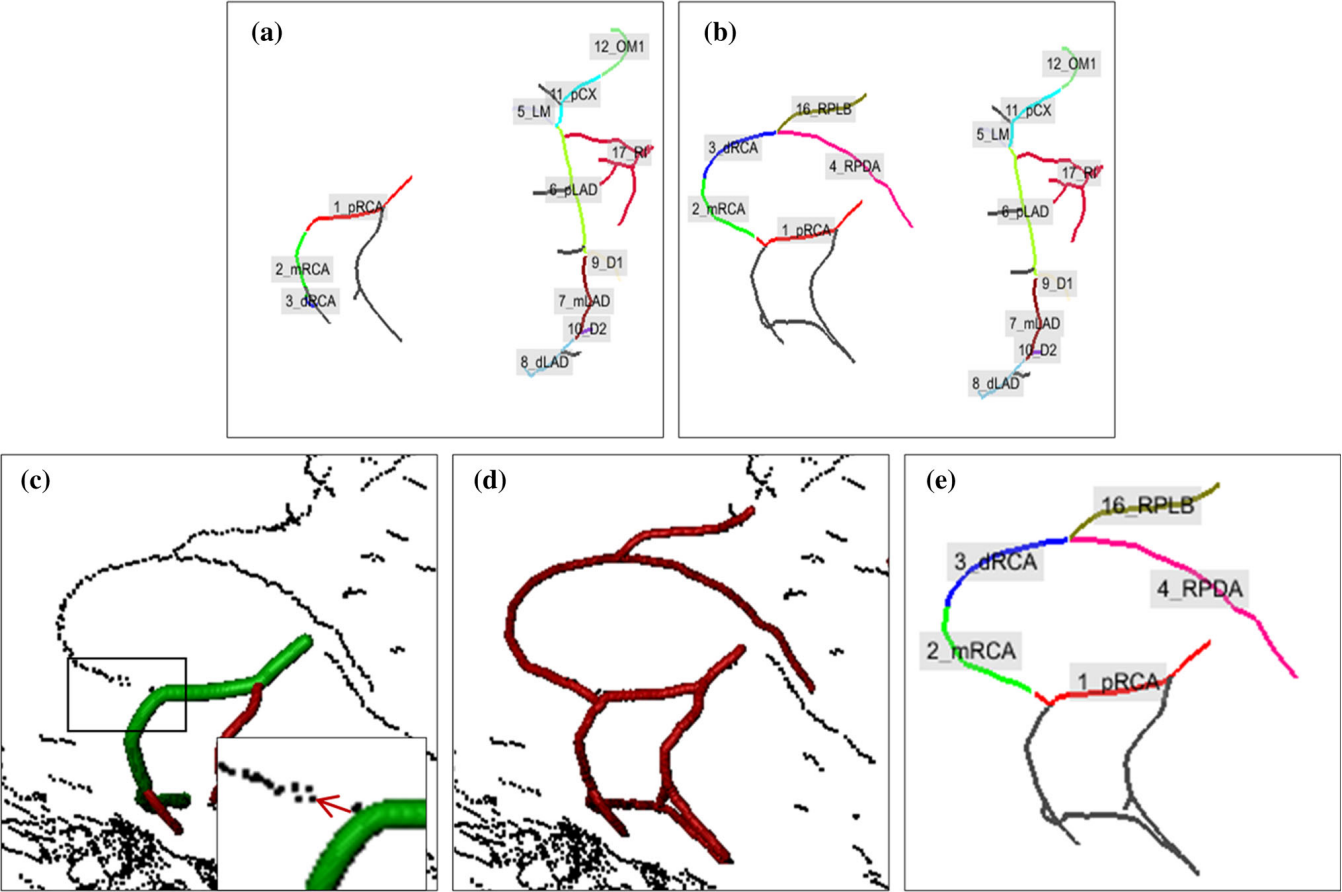
Automatic improvement to get RPDA and RPLB for a RD case by extension. **a** Initially extracted CAT with labels. **b** An automatically improved CAT. **c**–**e** Zoom-in image of the sub-tree RCA. **c** Initially extracted RCA on the binary vesselness image; the green part shows the labeled segments and the red part shows the unlabeled segments; the box shows a zoom-in image of searching area, and the red arrow points out the extension direction. **d** Automatically extended RCA (red) on the binary vesselness image. **e** Extended RCA with labels. *RPDA* right posterior descending artery, *RPLB* right posterior lateral branch, *RD* right dominant, *CAT* coronary artery tree, *RCA* right coronary artery

From the automatically selected start points, unconnected vessel-like structures are searched iteratively starting with an initial searching distance and increasing by a step in each iteration. The search continues until unconnected parts are found or the maximum search distance is reached. Figure 4 shows an example of an extension operation for a RD case.

### The decision tree

**Fig. 5:**
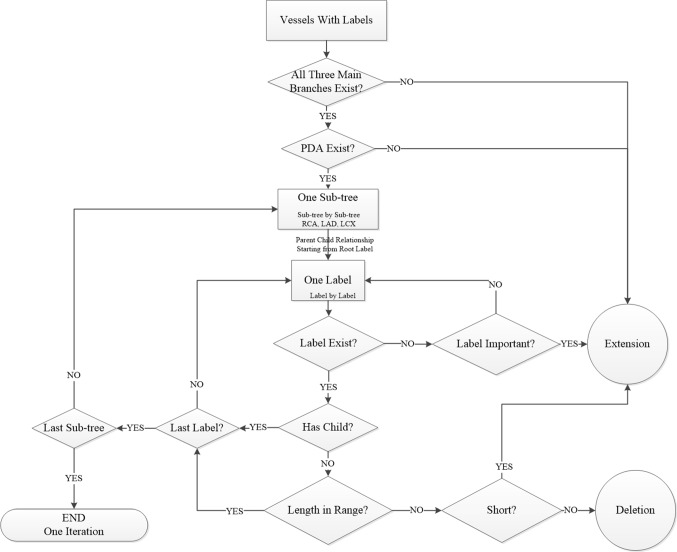
The decision tree for the improvement process. *PDA* posterior descending artery, *RCA* right coronary artery, *LAD* left anterior descending artery, *LCX* left circumflex artery

Since different operations might be needed for the overall improvement in a CAT, the decision tree in Fig. 5 shows the order of improvement operations according to the importance of a label in the topology of the CAT and its influence on successive steps.

The top level detects the three main branches. If any of the three main branches is absent, the improvement operation will be performed until all of them are extracted.

The next level evaluates the dominance type since it determines which anatomical model to use. We use the automatic method proposed by Cao et al. [9] to decide the dominance type. However, their method determines a CAT as RD when both the LCX and RCA are not fully extracted. Therefore, the automatically determined dominance type is evaluated by checking the presence and correctness of the PDA since in the RD case it should be present in the RCA while in the LD case it should be present in the LCX [10]. If the PDA is absent or its length is incorrect, the corresponding improvement operation is performed until it is found and has a correct length.

The remaining levels of the decision tree are as follows. Based upon the three main branches (RCA, LAD and LCX), the CAT is split into three sub-trees. Each sub-tree contains one main branch and several side branches (Fig. 2). The improvement process will traverse sub-tree by sub-tree. In each sub-tree, the process starts from the root label to the leaf label. For each label, its presence, length and direction is evaluated and improved if necessary. If the current label has a child which is also present, the improvement step will move forward until reaching a leaf label. For example, if the mLAD is present and its child dLAD is also present, the improvement process checks the correctness of dLAD instead of the mLAD. If all labels in the current sub-tree are evaluated and improved, the process moves to the next sub-tree.

The improvement for all sub-trees, which might include several improvement operations, is counted as one iteration. The improvement process is performed iteratively in order to achieve a CAT with a better quality. This requires a quality measurement for evaluation and some criteria to stop the iterative process.

### Stopping criteria

A quality score for a CAT is calculated using the scoring system proposed by Cao et al. [9]. The quality scores before and after an improvement operation are represented as *S*_old_ and *S*_new_ respectively and $S_{\Delta } = (S_{\text{new}} - S_{\text{old}} )$ describes the change in quality after one improvement operation. If $S_{\Delta } > 0$, the improvement operation increases the quality of the CAT and the process moves to the next step. The improvement process stops when $S_{\Delta } = 0$ in one iteration.

If $S_{\Delta } < 0$, the improvement operation deteriorates the extracted CAT. To constrain the deterioration, a threshold $T_{S\Delta } \left( {T_{S\Delta } < 0} \right)$ is used. If $S_{\Delta } > T_{S\Delta }$, the current improvement operation is kept since a small quality score decrease could be improved by a later improvement operation. If $S_{\Delta } < T_{S\Delta }$, the current improvement operation will be reverted. Moreover, this improvement operation is skipped and the improvement process moves to the next step. After each step, the improved CAT is stored so the process can be reverted. The value of the $T_{S\Delta }$ is determined by a training process which can be found in “Supplementary Material Section 3.”

To prevent the extraction quality from continuously decreasing within the threshold ($S_{\Delta } > T_{S\Delta }$) after each improvement operation, the accumulated score difference $\sum S_{\Delta }$ is computed and when $\sum S_{\Delta } < T_{S\Delta }$, the automatic improvement process stops.

Additionally, a maximum number of iterations is set to prevent the improvement process from over-extracting artery-like structures due to unforeseen circumstances. The intermediate results of the improvements are stored and the extraction with the highest score will be the final extraction.

### Statistical analysis

Descriptive data are expressed as the average ± standard deviation (SD). The correlation between the quality score of the initial extraction and the score difference after automatic improvement is evaluated using Spearman correlation coefficient. The statistical computations were performed in SPSS (Version 20.0; IBM Corp, Armonk, NY). A 2-tailed *P* value less than 0.05 was considered to be statistically significant.

## Experiments and results

### Experiments

The performance of the proposed algorithm was assessed on two cohorts. The first cohort consists of 42 datasets (No. 0-41) from the MICCAI segmentation challenge workshop^1^ [14] which were distributed over five calcium categories to have a representative population for undergoing CTA examination. Patients with pacemaker or CTA of non-diagnostic image quality, such as motion artifacts, were excluded.

The second cohort consists of 98 cases (60 RD and 38 LD). The average image and voxel size of the datasets was 512 × 512 × 512 and 0.307 × 0.307 × 0.25 mm, respectively. These cases did not include severe lesions at the proximal part of main branches or coronary anomalies.

The initial CATs for all cases were extracted using the method of Yang et al. [6]. The model-guided method is applied to improve these initial CATs. To set up references for the proposed method, experts manually corrected all initial CATs if necessary. The first 18 datasets (No. 0-17) from the first cohort were used to optimize parameters (see the results in “Supplementary Material Section 4”). The remaining 24 datasets from the first cohort were used to test the performance of the proposed method using these settings. The proposed method is applied on the other cohort (98 cases) to evaluate its performance in a different cohort.

### Results

The proposed method was implemented in MeVisLab-2.7.1. In general, improving a CAT takes less than 2 min on a typical workstation with a 2.67 GHz Intel quad-core processor.

In total 122 cases, 24 testing cases from the first cohort and 98 cases from the second cohort were used to validate the proposed method. For all 122 automatically extracted CATs, the model-guided method was able to improve their average quality score from 87 ± 6 to 93 ± 4.

### MICCAI challenge cohort

For 24 (No. 18-41) cases from the first cohort, quality scores for the initially extracted, manually improved and automatically improved CATs are listed in Table 1. The developed model-guided method improved the 24 initial CATs to an average quality score of 93 ± 5. It shows 21 CATs were improved with a quality score increase of at least 1. The remaining 3 (12%) showed no score change indicating no improvement. More descriptions can be found in “Supplementary Material Section 5.”

**Table 1:** Quality scores of the initially extracted, manually improved and automatically improved coronary artery tree extractions for 24 testing cases from the first cohort

Case no.	Quality score				
	Initial extraction	Manual improvement	Automatic improvement	DIF (Manual-Init)	DIF (Auto-Init)
18	76	81	78	5	2
19	77	85	81	8	4
20	88	86	96	− 2	8
21	94	97	97	3	3
22	90	90	91	0	1
23	84	89	96	5	12
24	94	94	95	0	1
25	94	93	96	− 1	2
26	97	97	98	0	1
27	93	94	95	1	2
28	95	95	95	0	**0**
29	92	95	93	3	1
30	81	95	86	14	5
31	76	81	88	5	12
32	88	93	97	5	9
33	87	90	92	3	5
34	83	90	89	7	6
35	91	88	92	− 3	1
36	87	94	91	7	4
37	96	93	96	− 3	**0**
38	96	96	96	0	**0**
39	90	95	95	5	5
40	92	95	96	3	4
41	82	82	92	0	10
Min	76	81	78	− 3	0
Max	97	97	98	14	12
Median	90	93	95	2	3
Average (± SD)	88 (± 6)	91 (± 5)	93 (± 5)	3 (± 4)	4 (± 4)

Bold numbers are cases with same score after automatic improvement. DIF represents the difference in the quality score between the manually or automatically improved CAT and the initial CAT

*Min* minimum, *Max* maximum, *SD* standard deviation

### A different cohort for robustness

On the second cohort (98 cases), the robustness of the proposed method to different cohorts was measured. For the 98 cases, 89 initial CATs were automatically extracted without user interaction while the initial extraction for 9 (9%) cases failed due to a wide-field of view CCTA scanning. The initial CATs for these 9 cases were semi-automatically extracted with manually set aorta centers (6 cases) or ostia positions (3 cases).

**Fig. 6:**
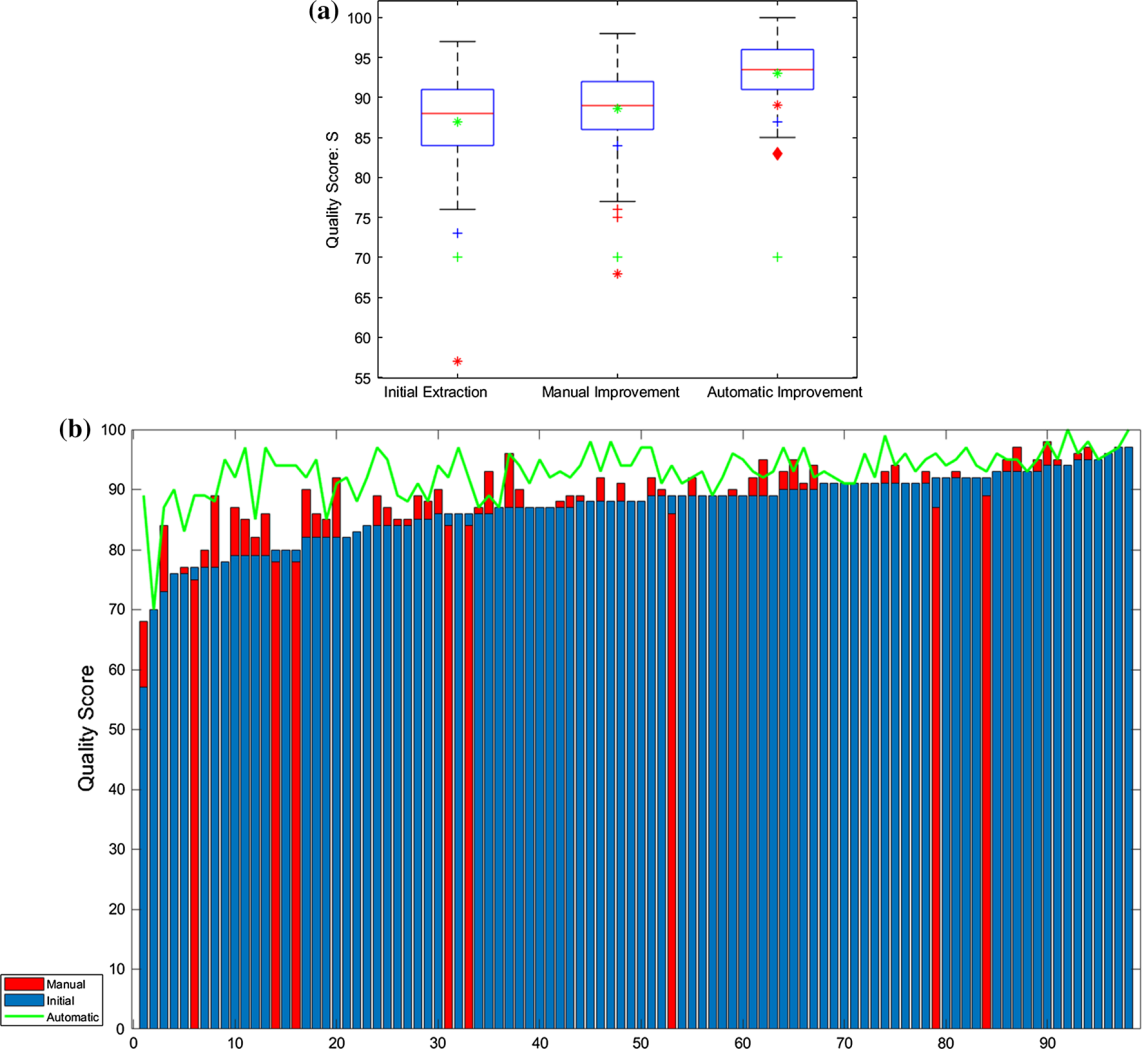
Quality scores of the initial extractions, manual improvements and automatic improvements. **a** Box-plots of the quality scores for initial extractions, manually improved and automatically improved extractions with their medians as 88, 89 and 94. Green * shows their average quality scores which are 87 ± 6, 89 ± 6, and 93 ± 4. For outliers, green + represents a case that the LM does not exist which is a coronary artery anomaly; red * represents a case that the LAD is not automatically extracted; blue + represents a RD case that only the proximal part of the RCA was extracted; red diamond represents a case that the distal part of the LCX could not be extracted. **b** Bar-plots of the quality scores sorted by the scores of the initial extractions. Red, blue bars show the scores for the manually improved and initial extractions; green line shows the scores of automatically improved extractions. *LM* left main artery, *LAD* left anterior artery, *LCX* left circumflex, *RD* right dominant

Quality scores of the automatically extracted, manually improved and automatically improved CATs for the 98 cases are shown in Fig. 6. Box-plots in Fig. 6a show the quality score distributions among 98 cases. Compared to the automatic extractions, all improved CATs have either the same (10 cases) or a higher quality score (88 cases). The average quality score for all 98 automatically improved CATs is 93 ± 4. Two cases had a final quality score of 100 after the automatic improvement, since they are the same as the designed anatomical statistical models. For all outliers in Fig. 6a, there is a detailed description in “Supplementary Materials Section 6.”

From left to right in Fig. 6b, the scores of the initial extractions are shown from small to large. The difference among the scores from the initial extraction, manual improvement and automatic improvement can be seen from the *y*-axis. In general, the proposed method improves the initial extraction in a similar manner as the manual improvement.

**Fig. 7:**
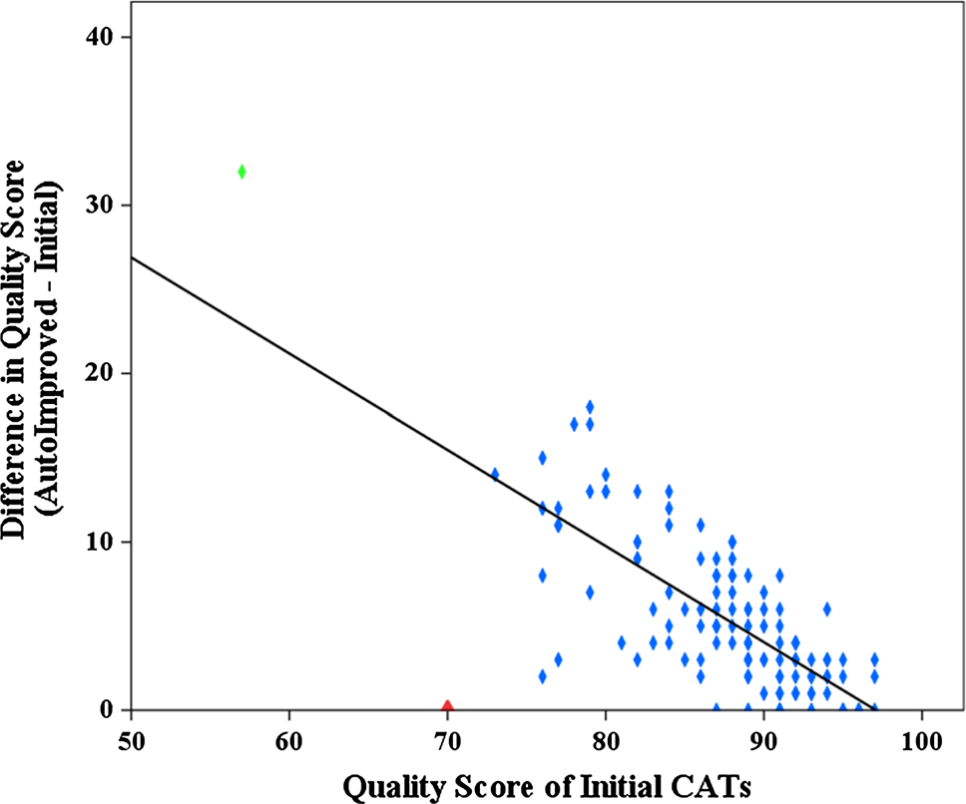
Correlation between the quality score of initial CATs and the difference in quality scores (*R* = − 0.694, *P* < 0.001) for 122 cases. The difference in quality scores is (the score of automatically improved CAT—the score of the initial CAT). Green diamond is the case with the LAD not automatically extracted. Red diamond is the case without LM. *CATs* coronary artery trees, *LAD* left anterior descending artery, *LM* left main

The proposed method improved the initial CATs for the first and second cohort to a similar average quality score (93 ± 5 vs 93 ± 4), and median quality score (95 vs 94). For all 122 cases, Spearman correlation analysis was performed between the initial quality score and the difference in score after automatic improvement and the result is provided in Fig. 7. There is a strong, negative correlation between the difference in score and the initial quality score (*R *= − 0.694, *P* < 0.001). A relatively small initial quality score implies a poor initial extraction, and therefore the automatic improvement increases the extraction quality much more than the cases where the initial score is already average.

To further assess the performance of the proposed model-guided method, we artificially pruned 5 fully extracted CATs and then applied the proposed method to improve them. The proposed method completely recovered the 5 artificially pruned CATs to their original CATs. We didn’t perform experiments for the remaining 93 cases, since it is to be expected that similar to the 5 selected cases, the proposed method is able to fully recover a CAT if there are continuous vessel-like structures in the binary vesselness image. Details of the automatic recovering process can be found in “Supplementary Material Section 7.”

## Discussion and conclusion

In this paper, an automatic method for improving coronary artery tree (CAT) extractions is described. Guided by anatomic statistical models, the proposed method is able to identify and improve incorrect extractions for automatically extracted CATs without deterioration. Although the initial extractions and binary vesselness images were extracted using a specific extraction method, the presented approach could also be applied to improve CATs extracted by other methods which can produce a candidate-list of vessel-like structures [2–5]. Automatically correcting CATs will reduce or even avoid the manual corrections for the radiologists or cardiologists. By providing a precise and complete CAT, the model-guided method will also help clinicians save time in performing analysis. Furthermore, automatically improved CATs will not introduce manual bias which are more reproducible. The proposed method can be safely applied and may facilitate the automatic analysis of coronary artery disease.

### Searching distance

The improved branch searching is performed with an optimally selected initial searching distance and increasing step-size. If the searching distance is too small, no vessel-like structures are found; if the searching distance is too large, a lot of vessel-like structures are connected to the CAT and the search takes more time. Additionally, the presented method is able to improve the extracted CAT for cases with chronic total occlusion with a lesion length shorter than the maximum searching distance (15 mm).

### Weight of the label

Only the absence of a label with a high weight is treated as an incorrect extraction and the proposed method is applied to improve it. This reduces the searching time and the risk of including non-artery structures. The label weight threshold is empirically set as 0.4. Potentially, the miss-extractions on a low weight label could be missed while a miss-extraction on a high weight label could be over-extracted.

### Stopping criteria

We defined the stopping criteria since it is difficult to set a generic standard score to judge the quality of extracted CATs from different scans. The change of the quality score after each improvement operation indicates the performance of one improvement action. The score decreasing threshold is used to ensure that the improvement operations don’t deteriorate the quality of the CAT too much. However, we should also point out that some vessel-like structures might be included in the automatically improved results.

### Comparison with manual corrections

We also included the manually corrected CATs as a reference. In general, more vessels are included in the automatically improved CAT compared to the corresponding manually corrected CAT. The manual correction was focused on removing veins and vessel-crossings while the model-guided method improves the CAT by considering not only the removal of wrong extractions but also the extension to generate a more complete CAT.

Due to the large anatomical variation in CATs among the general population, it is not possible to define a generally applicable cut-off of the quality score to indicate which case needs corrections. Furthermore, it should be noted that improving a CAT to a score of 100 is not the goal of the proposed method since only a CAT with exactly the same topology as defined in the anatomical statistical model will achieve 100.

### Limitations

For vessels not in the AHA model, only corrections on the pathline length and vessel directions could be made. Additional features, such as anatomical locations, should be exploited. Also, the proposed method could not be performed when the initial extraction failed due to a wide-field of view CCTA scanning. It is to be expected that initial extractions will be successful by cropping the CCTA images around the heart which will improve the performance of the proposed method. Finally, no comparison between the improved coronary artery trees and the results from the experts was made due to the difficulty in obtaining ground-truth manual extractions from experts. Experts may focus on different aspects of a CAT or may have a different purpose with the extracted trees.

## Electronic supplementary material

Below is the link to the electronic supplementary material.

Supplementary material 1 (DOCX 1212 kb)

## Footnotes

1 http://coronary.bigr.nl/stenoses/about.php.
