# Supplementary material for: A model-guided method for improving coronary artery tree extractions from CCTA images
Source: Int J Comput Assist Radiol Surg. 2018 Nov 28;14(2):373–83. doi: 10.1007/s11548-018-1891-7 (PMC6373332; doi:10.1007/s11548-018-1891-7)
Supplement: Supplementary file 1 — Supplementary material 1 (DOCX 1212 kb) [file 11548_2018_1891_MOESM1_ESM.docx]

Supplemental Material for the Article:

A Model-guided Method for Improving Coronary Artery Tree Extractions from CCTA images

Qing Cao^1^, Alexander Broersen^1^, Pieter H. Kitslaar^1,2^, Boudewijn P.F. Lelieveldt^1^, Jouke Dijkstra^1^

1. Division of Image Processing, Department of Radiology, Leiden University Medical Center, Leiden, The Netherlands

2. Medis medical imaging systems bv, Leiden, The Netherlands

## Anatomical statistical models

The weight for each label in the 17-segments AHA model based on the occurrence of each label to describe its clinical significance (Fig. 1).

| 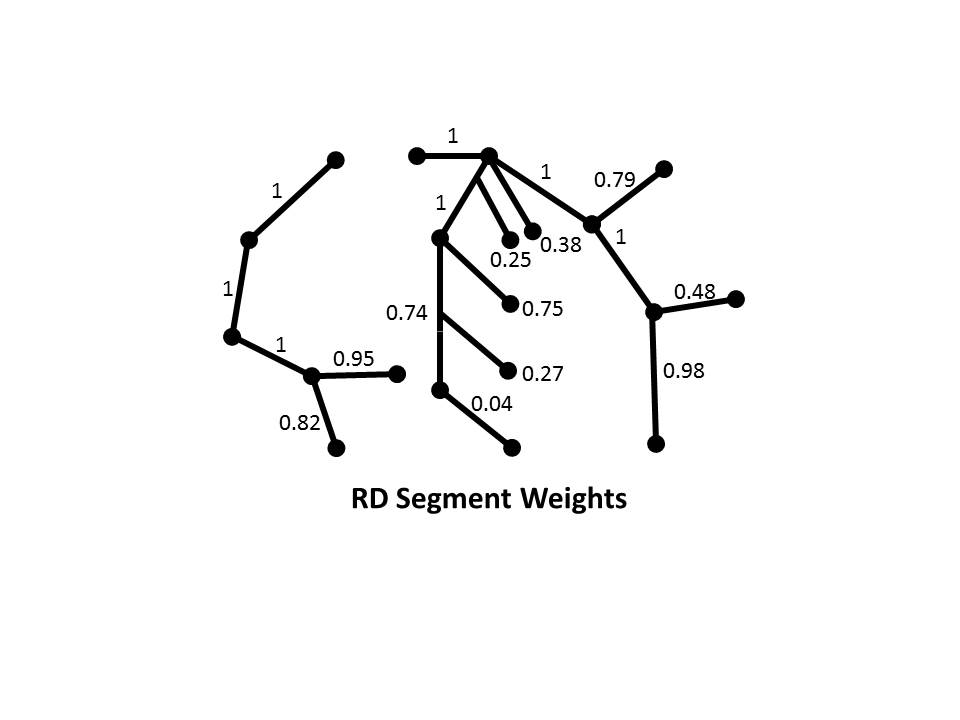 | 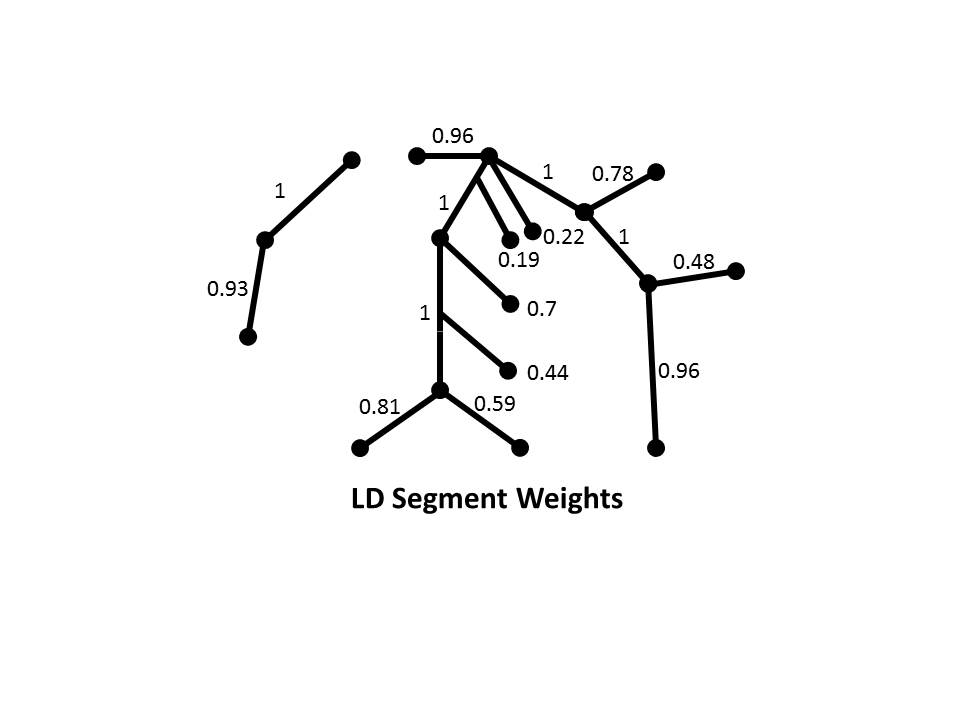 |
| --- | --- |
| (a) | (b) |

1. The weight for each label in the 17-segments AHA model for both RD and LD cases. LD= Left Dominant; RD= Right Dominant

## Coarse Improvement

| 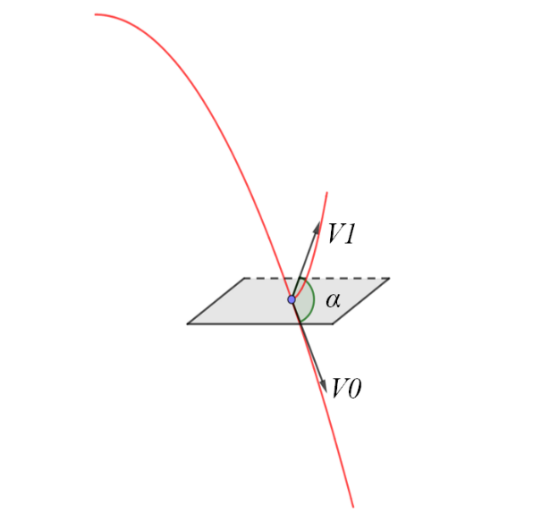 | 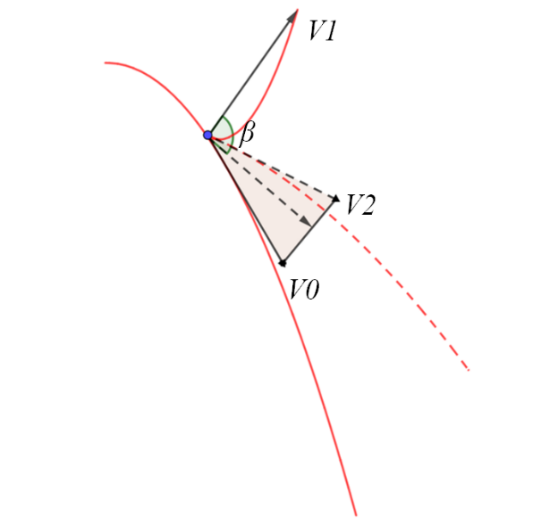 |
| --- | --- |
| (a) | (b) |

1. The bifurcation angle *α* and the surface angle *β* of a vessel. (a) The bifurcation angle *α* of a vessel between vessel direction vector *V1* and the main branch direction vector *V0*. (b) The surface angle *β* between vessel direction vector *V1* and the plane defined by the main branch direction *V0* and the other vessel direction vector *V2*

Two angles are involved in the coarse improvement process. Along the blood flow direction, the angle between the parent branch direction *V0* and the side branch direction *V1* is calculated as the bifurcation angle *α* of this side branch (Fig. 2a). When *α*>120°, the vessel is removed since it indicates a sudden change in direction. If multiple vessels bifurcate from the same position on a branch, the surface angle *β* of a side branch is calculated as the angle between *V1* and the plane defined by *V0* and *V2* (the other vessel direction) (Fig. 2b). Vessels with *β*>120° are removed.

Fig. 3 shows some examples of the coarse improvements applying on automatically extracted CATs. Blue lines show the extractions removed by coarse improvement operations.


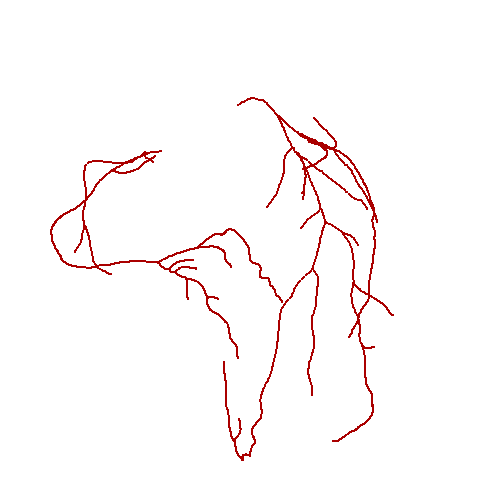

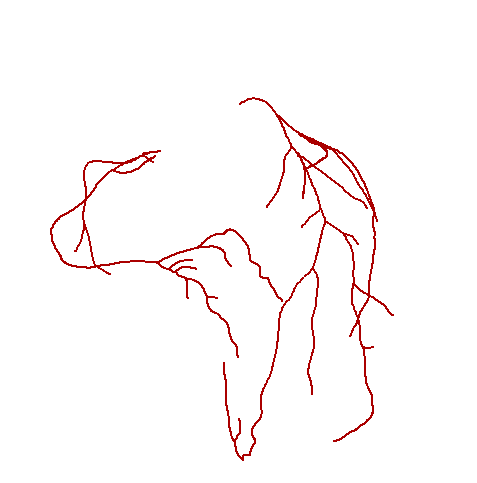

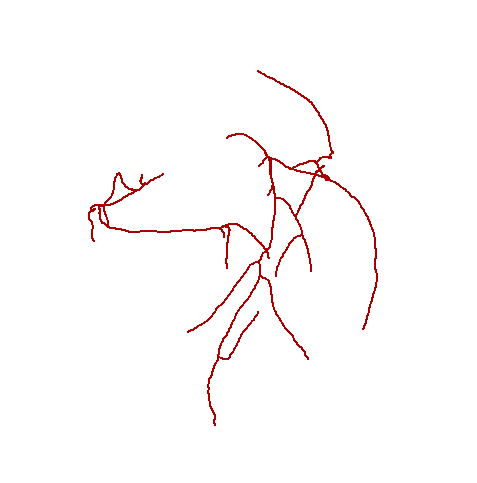

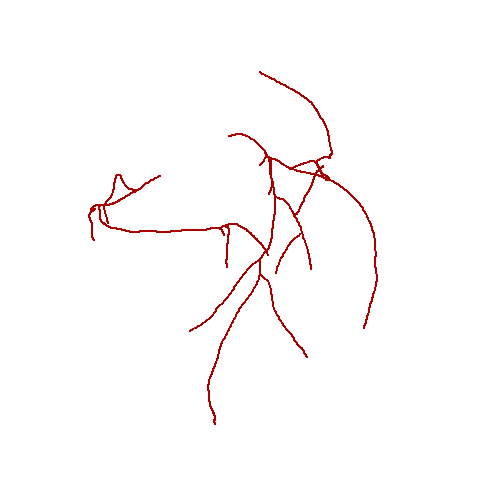

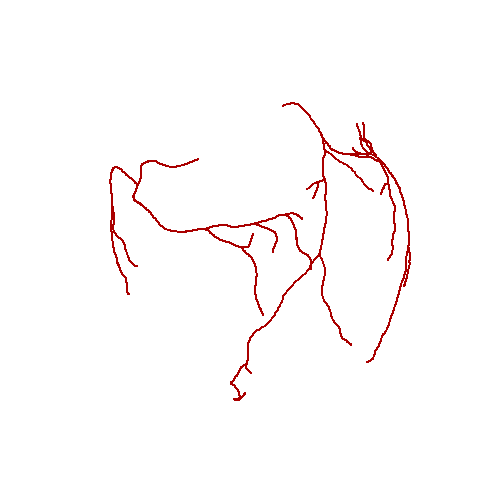

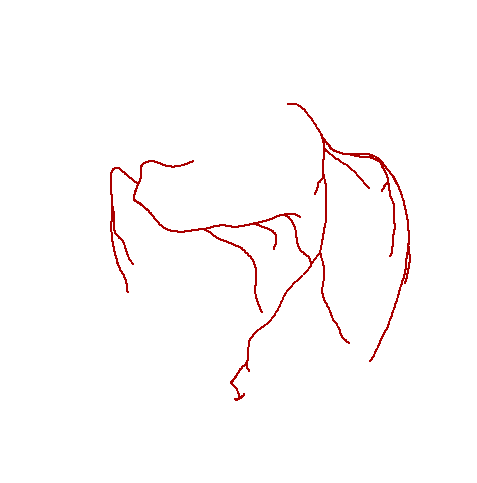

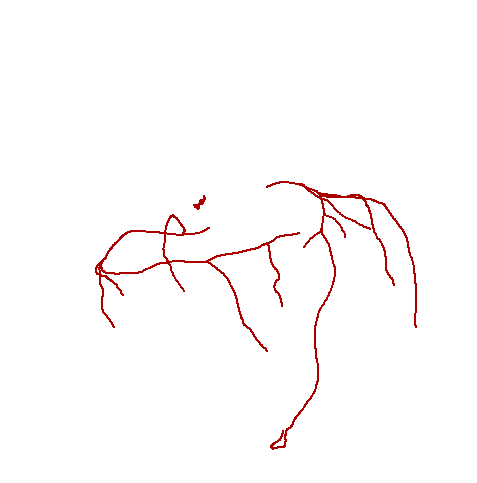

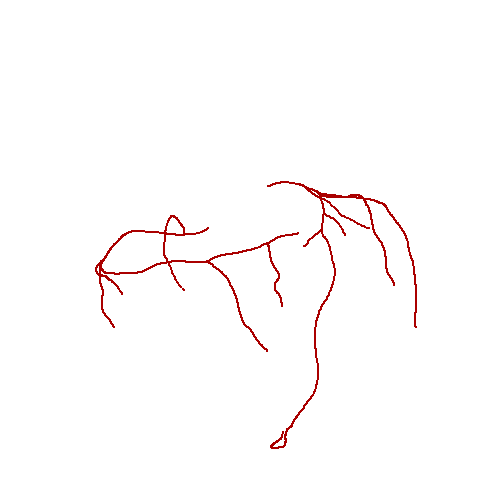

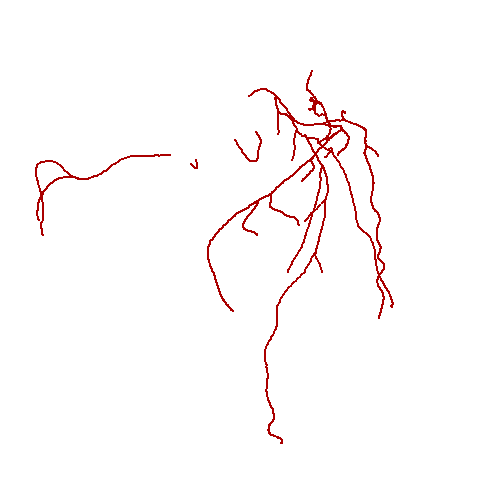

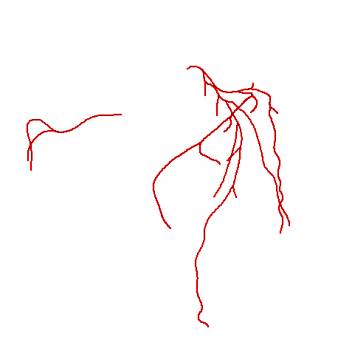

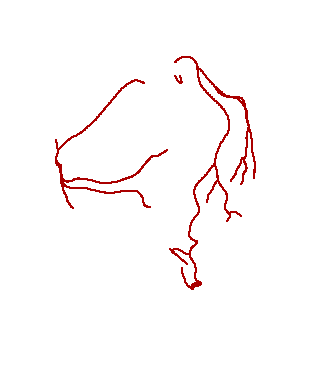

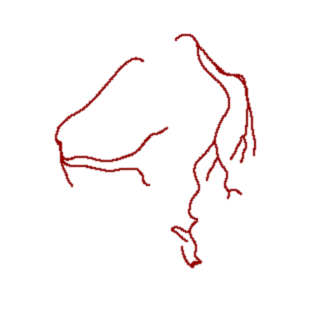


1. Coarsely improved 6 CATs. Blue lines are the vessel-like structures removed by coarse improvement operations.

## Sensitivity Analysis of the Parameters

The proposed method involves four parameters, namely, *l_0_* and *µ* to initiate, increase and constrain the size of the searching distance; *T_S∆_* as a threshold to limit the quality score decrease and the maximum number of the iterations *N_iter_*. The first 18 datasets (No. 0-17) from the first cohort were used to optimize all 4 parameters. Their values are listed in Table 1 with which the highest average score was achieved.

1. Parameters setting for the model-guided improvement method

| Parameters | Value |
| --- | --- |
| *l_0_* (mm) | 6 |
| *µ* (mm) | 2.5 |
| *T_S∆_* | -4 |
| *N_iter_* | 4 |
| *T_weight_* | 0.4 |

For a fast and accurate branch searching, the initial searching distance *l_0_* is set as 6 mm after training on 18 training sets. The step *µ* is set as 2.5 mm to allow some cases with larger gaps. In practice, a small initial searching distance (e.g., *l_0_*=2 mm) can be used and increased by a step size *µ=*1 mm in case of including too many non-artery structures in the CAT which is a trade-off with time. A maximum searching distance of *L_max_*=15 mm is still set to avoid extracting surrounding tissues as arteries. Moreover, if there is no limitation for the *L*, the searching time and also the risk of extracting surrounding tissues or veins as arteries increase. Absent labels in extracted CATs with weights larger than a threshold *T_weight_* are marked as important and need to be improved. *T_weight_* is set to 0.4 with which all main branch segments, the first marginal (OM1) and the first diagonal (D1) are considered as important.

To evaluate the performance of the proposed method, different starting points, searching distances, quality score thresholds and number of iterations are explored and their derived improvement results are analyzed.

- 1. The selection of different start points

Fig. 4 illustrates the process of extending from different start points to improve an extracted CAT. Firstly, the extracted CAT (Fig. 4a) is automatically determined as a RD case. Then, the labels in the AHA model are automatically assigned to corresponding segments in the extracted CAT as shown in Fig. 4d. After the comparison with the RD anatomical model, the absence of RPLB is detected. The extension operation is performed since the RPLB with a weight of 0.95 is important as being a RD case.

The points on the parent label (dRCA) of RPLB are selected as starting points for extension as described in the methods part. The RPLB is extracted and shown in Fig. 4b and e. To illustrate the extension differences in selecting different start points, Fig.4c is the result of the extension using all points on the RCA as starting points. Even though the RPLB is extracted in Fig. 4c, several non-vessel structures are also included in the RCA (pointed out by blue arrows). Moreover, if more points are used as starting points, the searching time increases compared with the extension only from the dRCA. For better understanding, the second row from Fig. 4d-f show the CATs with labels corresponding to the CATs in the first row (Fig. 4a-c).

| 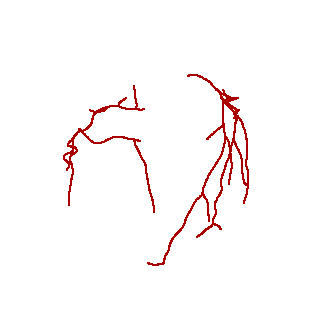  (a)  (b)  (c) | 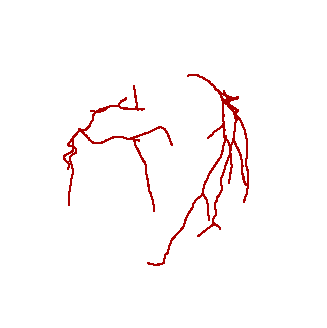 | 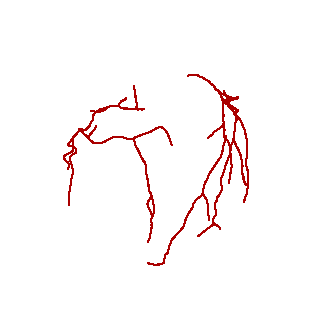 |
| --- | --- | --- |
| Coarsely Improved CAT  *S* =88.0 | *S_new_* =92.3; *S_old_*=88.0;  ***S_∆_*=4.3** | *S_new_* =90.9; *S_old_*=88.0;  ***S_∆_*=2.9** |
| 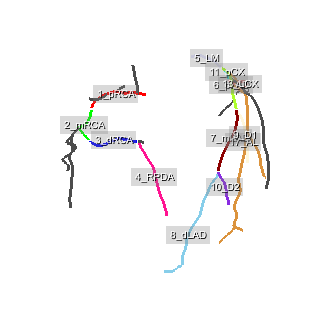  (d)  (e)  (f) | 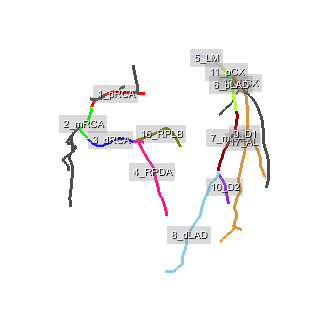 | 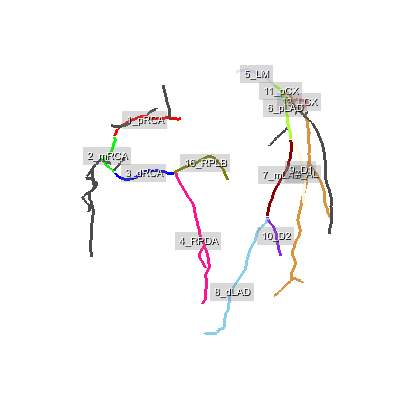 |

1. Extending from different starting points to get the RPLB. (a) The coarsely improved CAT. (b) The improved CAT extending from the dRCA. (c) The improved CAT extending from the whole RCA. *S_new_*, *S_old_* and *S_∆_* are the quality scores after one improvement operation, before one improvement operation, and also their difference. The second row (d-f) shows the CATs with labels corresponding to the CATs in the first row (a-c). RPLB= Right Posterior Lateral Branch; CAT= Coronary Artery Tree; RCA= Right Coronary Artery; dRCA= distal Right Coronary Artery.

Different from the approach to extend a side branch, if a main branch label is absent, all points with large curvatures on the extracted part of the main branch are used as starting points. Fig. 5 shows an example of the extension to get the RPDA and RPLB for a RD case. Fig. 5a and d show a coarsely improved CAT in which the dRCA is assigned to an incorrect position since the real dRCA is not extracted. If the branch searching starts from segment dRCA (the part between the two red points in Fig. 5d), the real RCA branch cannot be extracted, as can be seen from Fig. 5b and e. A search from the whole RCA will successfully extract the real RCA branch (Fig. 5c and f).

| 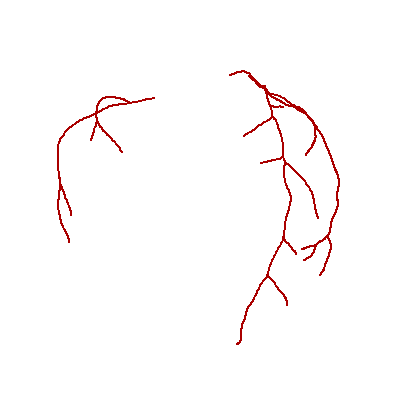  (a)  (b)  (c) | 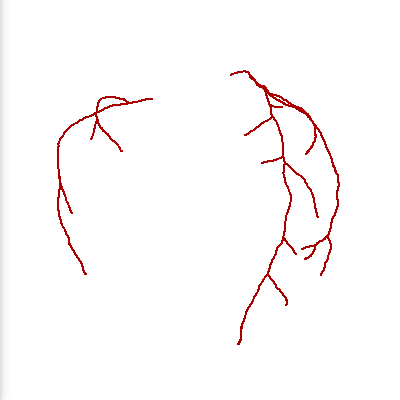 | 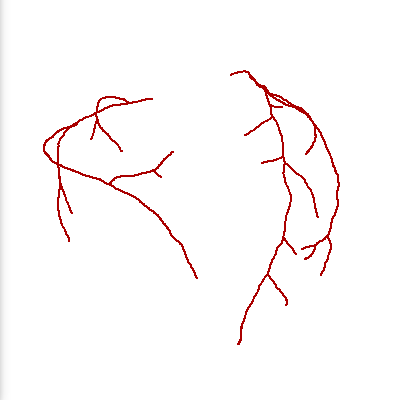 |
| --- | --- | --- |
| Coarsely improved CAT | Extension from the dRCA | Extension from the whole RCA |
| 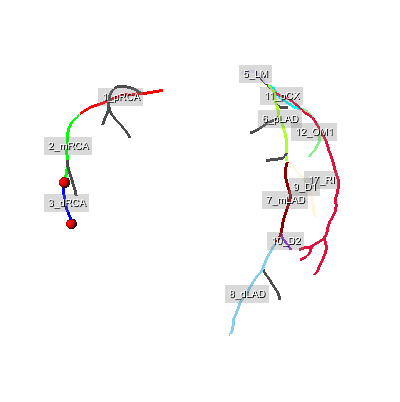  (d)  (e)  (f) | 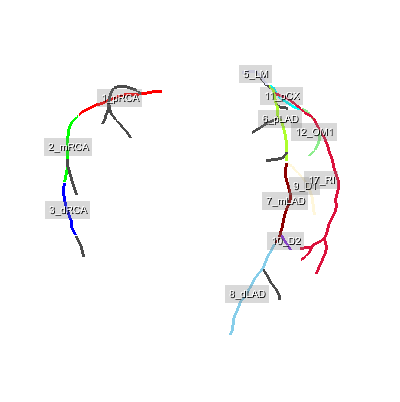 | 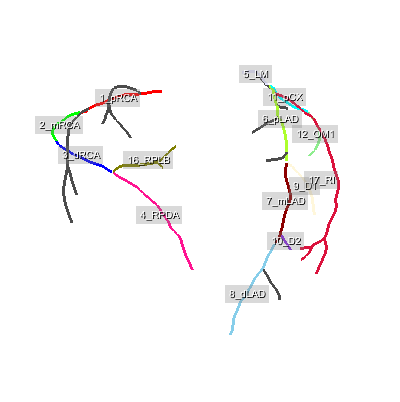 |

1. Extending from the main branch to get the RPDA and RPLB for a RD case. (a) The coarsely improved CAT. (b) The improved CAT extending from the dRCA. (c) The improved CAT extending from the whole RCA. The second row (d-f) shows the CATs with labels corresponding to the CATs in the first row (a-c); the two red points in (d) show the start and end points of the segment dRCA. RPDA= Right Posterior Descending Artery; RPLB= Right Posterior Lateral Branch; RD= Right Dominant; CAT= Coronary Artery Tree; RCA= Right Coronary Artery; dRCA= distal Right Coronary Artery.

Fig. 6 shows another example of automatically searching for D1 and the difference between extending from the pLAD and the whole LAD. On the coarsely improved CAT (Fig. 6a and d), the proposed method searches from the points on the pLAD (pointed out by two red points in Fig. 6d to find the D1. One vessel-like branch is added (the blue arrow in Fig. 6b) after the extension which is not the wanted D1, as can be seen from Fig. 6e. However, the quality score remains the same since the added one branch have small effects on the quality score. Searching from the whole LAD results in extracting more unwanted vessel-like structures as arteries, seen in Fig. 6c and f. These added structures affect the topology of the CAT leading to an incorrect automatic labeling for the CAT in Fig. 6f.


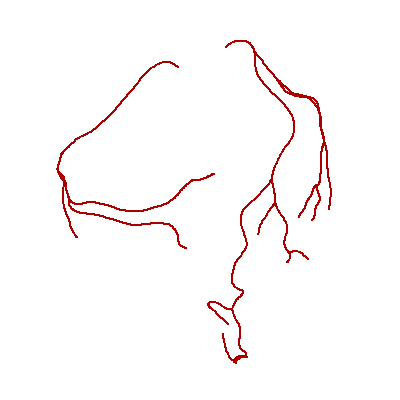

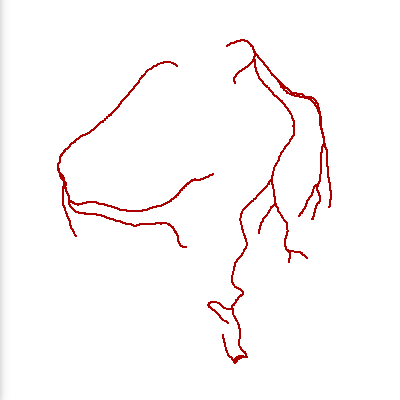

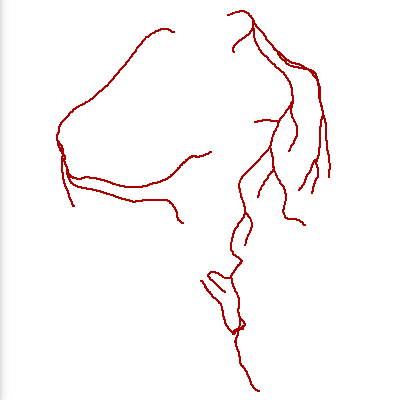


Coarsely Improved CAT

*S* =89.6

*S_new_* =89.6;

*S1*=86.9;*S2*=100.0

*S_old_*=89.6; ***S_∆_*=0**

*S_new_*=85.6;

*S1*=78.3;*S2*=63.7

*S_old_*=75.3; ***S_∆_*=-10.3**

(a)

(b)

(c)


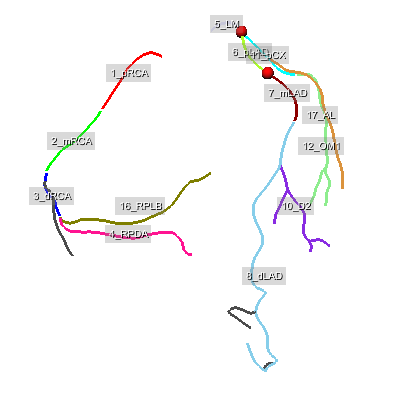

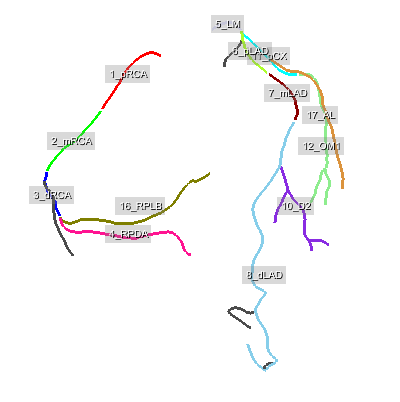

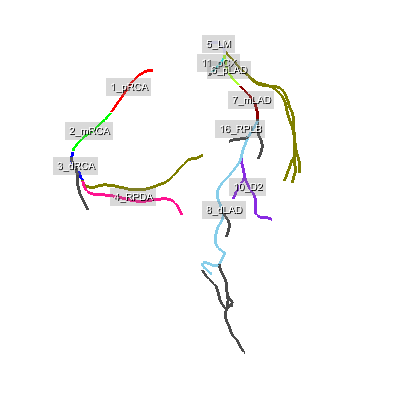


(d)

(e)

(f)

1. Extending from different start points to get the D1. (a) The coarsely improved CAT. (b) The improved CAT extending from the pLAD with one vessel-like structure added; the blue arrow points at the added branch which is not the D1 branch. (c) The improved CAT extending from the whole LAD with too many incorrect extractions added; blue arrows point at the added branches which are not the D1. The second row (d-f) shows the CATs with labels corresponding to the CATs in the first row (a-c); the two red points in (d) point at the segment pLAD. *S_new_*, *S_old_* and *S_∆_* are the quality scores after one improvement operation, before one improvement operation, and also their difference. *S1*, *S2* are the sub-quality scores for the *vessels-with-labels* and the *vessels-without-a-label*. The lower *S2*=63.7 from (c) compared to the *S2*=100.0 in (b) is caused by the added wrong extractions. D1= first Diagonal; CAT= Coronary Artery Tree; pLAD= proximal Left Anterior Descending branch.
   1. Searching distance

From the automatically selected starting points, unconnected vessel-like structures in the corresponding binary vesselness image are searched iteratively starting with an initial searching distance *L=l_0_* and increasing in each iteration with step *µ* (*L=L+µ*). The searching continues until unconnected parts are found or the maximum search distance *L_max_* is reached. If multiple unconnected parts are found, the unconnected part with the most and the longest side branches is selected and connected to the extracted CAT.

Fig. 7 shows the automatic improvement process for an extracted CAT extending with different searching distances. The initial CAT (Fig. 7a and e) is automatically determined as a RD case. A coarse improvement is first performed to remove the vessels with wrong directions which are pointed out by two blue arrows in Fig. 7a. A further detection reports that an extension of the RCA to get the RPDA and RPLB is needed for this RD case. As can be seen from Fig. 7, extending with different searching distances from the same start points, different results will be obtained.

When the searching distance *L*<5 mm, no vessel-like structures are found and connected (Fig. 7b and f). Fig. 7c and d show the result of searching with *L*=6 mm which added both the RPDA and RPLB. When *L*=12 mm, the RPDA and RPLB are extracted while some unwanted vessel-like structures are also connected to the CAT (see blue arrows in Fig. 7d). The score of the CAT in Fig. 7d is lower than Fig. 7c because additional *vessels-without-a-label* are included. Additionally, the searching takes more time.


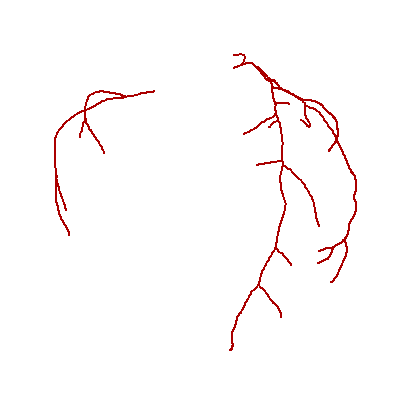

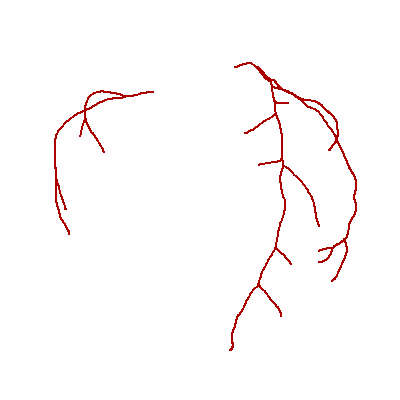

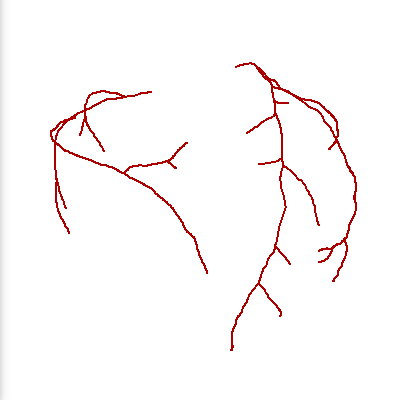

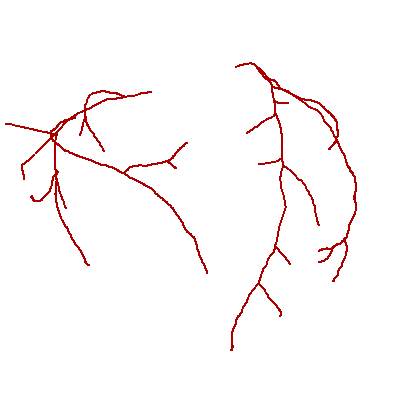


Initial CAT

*S*=86.0

*S_new_* =89.1; *S_old_*=86.0;

***S_∆_*=3.1;** *L***=3 mm**

*S_new_* =95.0; *S_old_*=89.1;

***S_∆_*=5.9**; *L***=6 mm**

*S_new_* =91.0; *S_old_*=89.1;

***S_∆_*=1.9;** *L***=12 mm**

(a)

(b)

(c)

(d)


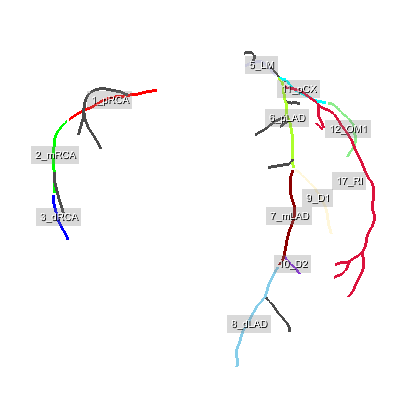

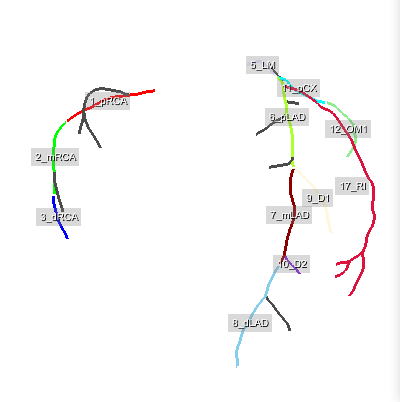

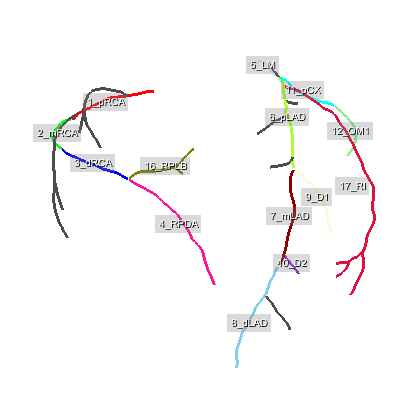

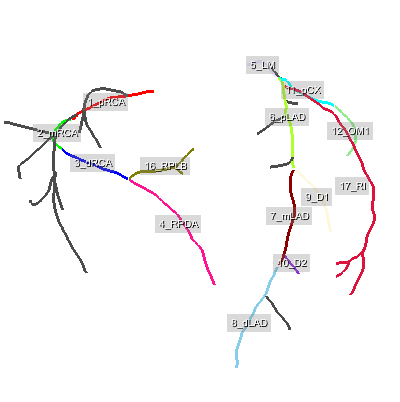


(e)

(f)

(g)

(h)

1. Extending the RCA with different searching distances. (a) Automatically extracted CAT; blue arrows point at vessels with wrong directions which will be removed by the coarse improvement before extending. (b) Extending the RCA for the coarsely improved CAT with the *L*=3 mm and no vessels are added. (c) Extending the RCA with *L*= 6 mm and two vessels are added; blue arrows point at the added two branches: the RPDA and the RPLB. (d) Extending the RCA with *L*=12 mm and two vessels are added together with several additional vessel-like structures which are pointed out by blue arrows. The second row (e-h) shows the CATs with labels corresponding to the CATs in the first row (a-d). *S_new_*, *S_old_* and *S_∆_* are the quality scores after one improvement operation, before one improvement operation, and also their difference. RCA= Right Coronary Artery; CAT= Coronary Artery Tree; RPDA= Right Posterior Descending Artery; RPLB= Right Posterior Lateral Branch.
   1. Changes in quality scores

Fig. 8 shows the process of an extracted CAT improved by several improvement operations. The quality scores of the CAT after each improvement operation are calculated. In order to get the RPLB and RPDA, the extension of the RCA in Fig. 8b-d shows that the score decreased by 3.5, 0.3, and 7.3, respectively. Without the constraint for the changes in quality scores after each improvement operation or the accumulated score differences, the improvement process continuously worsen the extraction quality.


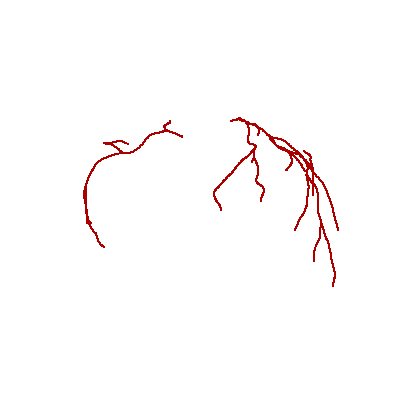

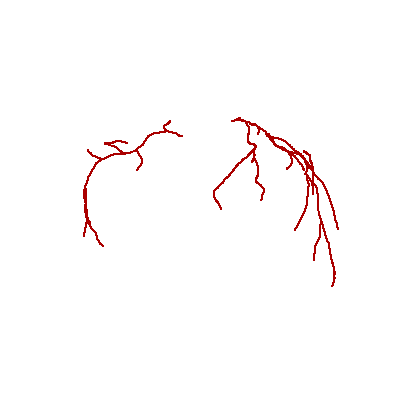

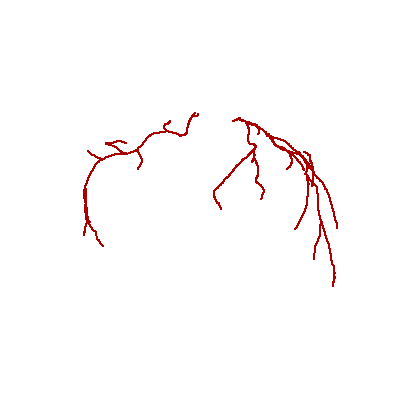

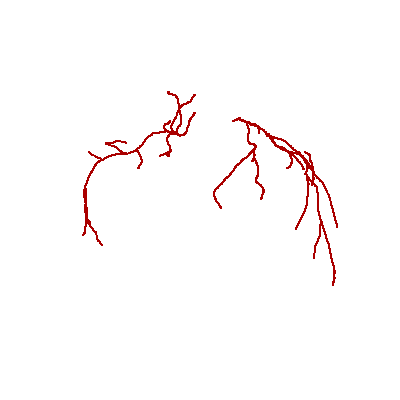


Coarsely Improved CAT

*S*=81.9

*S_new_* =78.4; *S_old_*=81.9;

***S_∆_*=-3.5;** **∑*S_∆_*=-3.5**

*S_new_* =78.1; *S_old_*=78.4;

***S_∆_*=-0.3**; **∑*S_∆_*=-3.8**

*S_new_* =70.8; *S_old_*=78.1;

***S_∆_*=-7.3; ∑*S_∆_*=-11.1**

(a)

(b)

(c)

(d)


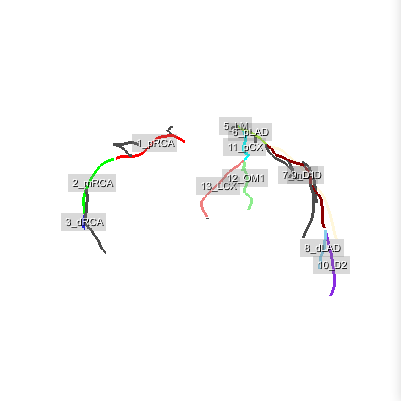

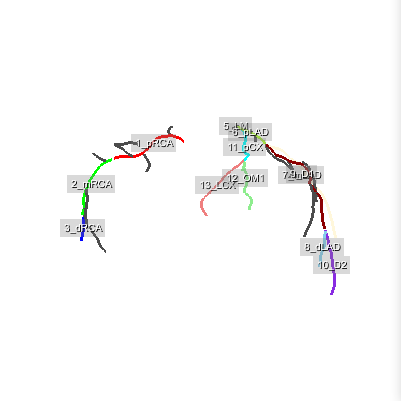

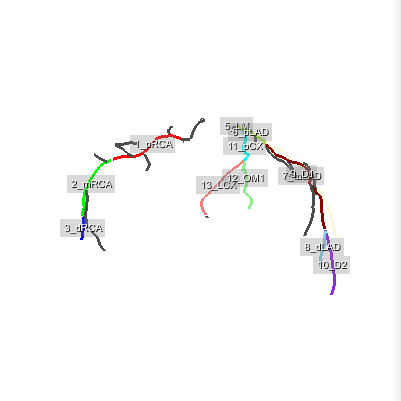

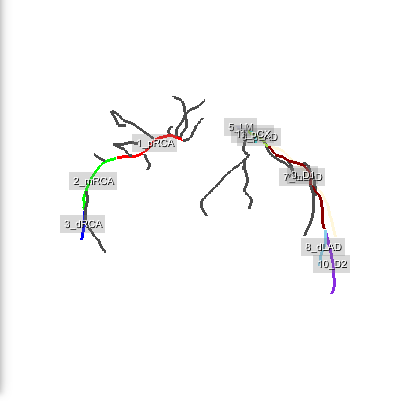


(e)

(f)

(g)

(h)

1. The changes in quality scores by several improvement operations to extend the RCA in order to extract RPDA and RPLB. (a) The coarsely improved CAT. (b-d) are the improved CATs extending for the first, second and third time, respectively. The RPDA and RPLB are not extracted while several unwanted vessel-like structures are added to the CATs (pointed out by blue arrows). The second row (e-h) shows the CATs with labels corresponding to the CATs in the first row (a-d). *S_new_*, *S_old_* and *S_∆_* are the quality scores after one improvement operation, before one improvement operation, and also their difference. *∑S_∆_* is the accumulated score difference during the improvement process. RCA= Right Coronary Artery; RPDA= Right Posterior Descending Artery; RPLB= Right Posterior Lateral Branch.
   1. The number of iterations

Fig. 9 shows the automatically improved results for an extracted CAT within different iterations. On the coarsely improved and labeled CAT (Fig. 9a and e), the absence of OM1 is detected which requires an extension. Points on the parent label (pCx) of the OM1 are used as starting points to do the extension (*l_0_*=6 mm; *µ* =2.5 mm). After doing the branch searching for 3 iterations, there were no vessel-like structures added to the LCX subtree (Fig. 9b). Actually, this case doesn’t have an OM1. Without the constraint on the number of iterations, the extension will not stop. After 6 and 9 iterations in the branch searching, some unwanted vessel-like structures are added to the LCX subtree which are pointed out by the blue arrows in Fig. 9c and d.

Coarsely improved CAT

***iter*=3**

***iter*=6**

***iter*=9**


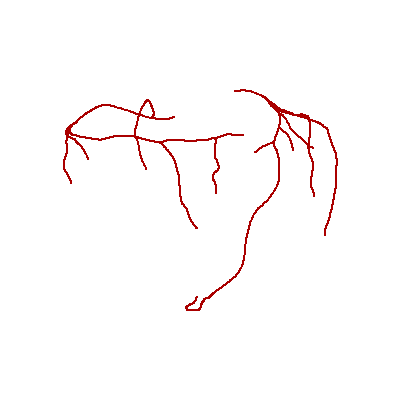

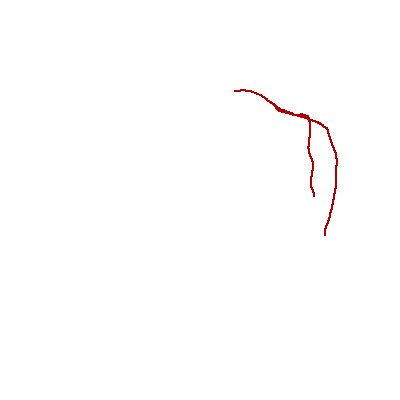

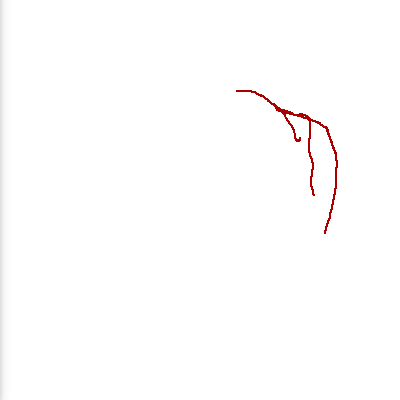

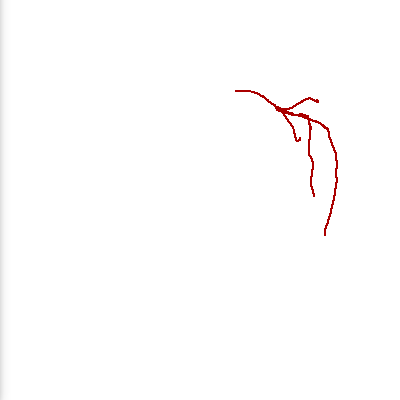


(b)

(c)

(d)

(a)


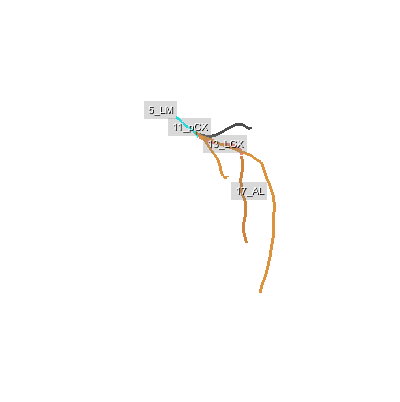

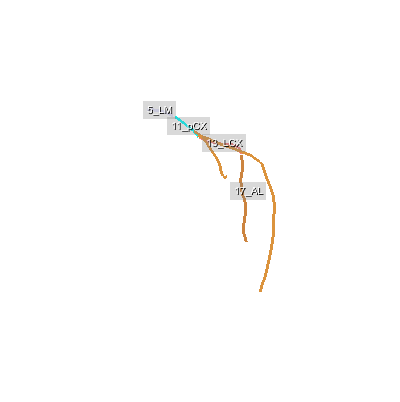

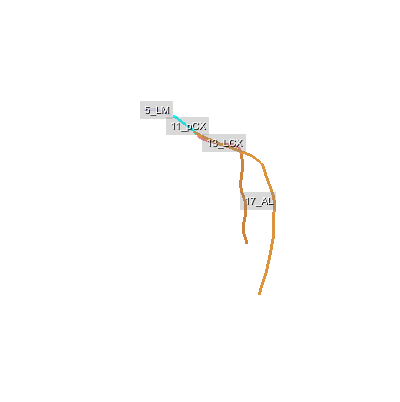

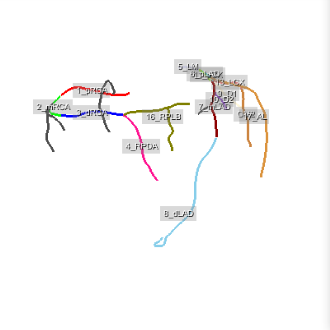


(f)

(g)

(h)

(e)

1. Extending from the pCx to get the OM1 within different iterations. (a) The coarsely improved CAT. (b-d) A close-up of the LCX subtree which shows the LCX subtree after extending in 3, 6 and 9 iterations, respectively; blue arrows point at the added branches. The second row (e-h) shows the CATs with labels corresponding to the CATs in the first row (a-d). pCx= proximal left Circumflex; OM1= first Obtuse Marginal; CAT= Coronary Artery tree; LCX= Left Circumflex.

## Results for the 18 training cases

Quality scores of the CATs from the 18 training cases before and after the automatic improvement are calculated and shown in Table 2. The average quality score of the 18 training datasets is improved from 84 to 93 with the standard deviation decreasing from 16 to 4.

1. Quality scores of the initially extracted, manually improved and automatically improved CATs for the 18 training cases (Case No. 0-17)

| Case No. | Quality Score | | | | |
| --- | --- | --- | --- | --- | --- |
|  | Initial  Extraction | Manual  Improvement | Automatic  Improvement | DIF  (Manual - Init) | DIF  (Auto-Init) |
| 0 | 88 | 88 | 93 | 0 | 5 |
| 1 | 87 | 91 | 88 | 4 | 1 |
| 2 | 22 | 92 | 95 | 71 | 73 |
| 3 | 89 | 86 | 97 | 0 | 8 |
| 4 | 77 | 84 | 86 | 7 | 9 |
| 5 | 88 | 88 | 89 | 0 | 1 |
| 6 | 75 | 74 | 85 | -1 | 10 |
| 7 | 93 | 88 | 95 | -5 | 2 |
| 8 | 90 | 92 | 100 | 2 | 10 |
| 9 | 92 | 99 | 99 | 7 | 7 |
| 10 | 92 | 91 | 96 | -1 | 4 |
| 11 | 86 | 92 | 95 | 6 | 9 |
| 12 | 86 | 87 | 95 | 1 | 9 |
| 13 | 91 | 85 | 96 | -6 | 5 |
| 14 | 84 | 80 | 86 | -4 | 2 |
| 15 | 89 | 81 | 97 | -8 | 8 |
| 16 | 86 | 90 | 91 | 4 | 5 |
| 17 | 89 | 89 | 97 | 0 | 8 |
| Min | 22 | 75 | 85 | -5 | 1 |
| Max | 93 | 97 | 100 | 74 | 73 |
| Median | 88 | 90 | 95 | 2 | 7 |
| Average  (±SD) | 84 (±16) | 90 (±5) | 93 (±4) | 6 (±17) | 10 (±16) |

Note: DIF represents the difference in the quality score between the manually or automatically improved CAT and the initial CAT. Min=Minimum; Max=Maximum; SD=Standard Deviation.

## Results of the MICCAI challenge cohort

In Fig. 6a of the main document, 3 initial CATs have *S*<75 which are shown as outliers. The outlier with *S*=70 is a case that the LM did not exist, which is a coronary artery anomaly (represented as a green + in Fig. 6a). This case is also shown as an outlier among the results from the manual improvement and automatic improvement. The case with the initial *S=*57 had its LAD not automatically extracted (represented as red * in Fig. 6a). The experts manually corrected this to a score of *S*=68. The proposed method automatically extended LAD and improved this CAT to *S*=89 which contributed to the maximum score increase *S_∆_*=32 among all datasets. The remaining outlier is a RD case for which only the proximal part of RCA was extracted which resulted in a lower score of *S*=73 (represented as blue + in Fig. 6a). The proposed method automatically added the remaining part of the RCA including the RPDA into this extracted CAT which results in a quality score of 87. In the automatically improved CATs, there is another outlier (*S*=83, represented as red diamond in Fig. 6a) except from the coronary artery anomaly case. In this case, a vein was located near the LCx in such a way that the vesselness filter could not distinguish between them and the LCx was detected as a vein. Due to lack of information in the binary vesselness image, our method couldn’t automatically extract the LCx.

## Description of the outliers in the second cohort

The last column of Table 1 in the main document displays the difference in quality score between the initial extractions and automatically improved CATs. The highest score increase (12) was for cases No.23 and No.31. For both cases, the dominance types could not automatically be determined since both the RCA and LCX were not completely extracted. For example, the “RD” dominance type is used initially in case No.23 but after the automatic improvement, it was successfully detected as LD.

## Automatic Tree Recovery

To further assess the performance of the proposed model-guided method, we artificially pruned 5 fully extracted CATs and then applied the proposed method to improve them. The proposed method completely recovered the 5 artificially pruned CATs to their initial CATs step by step based on the designed decisions tree. We show one of the 5 cases as an example (Fig. 10) to demonstrate the process of the model-guided method recovering an artificially pruned CAT to the initial fully extracted CAT.

A fully extracted CAT Fig. 10a is artificially pruned to the CAT in Fig. 10b by removing the RPDA, RPLB and D1 branch. The automatic tree recovery process has the following steps. Firstly, the dominance type of this case is automatically detected as RD. With the information that the RPDA is absent in this RD case, the RCA is selected to be extended. Branch searching is performed on the binary vesselness image from points on the RCA and the result is shown in Fig. 10c. The RPDA and RPLB were extracted in Fig. 10c with its quality score as 91. Later in the process, in the LAD subtree, the absence of D1 requires an extension due to its high weight. Points on the parent label pLAD of the D1 (Fig. 10c, marked as two red points) are used as searching starting points. The D1 is connected by the extension (Fig. 10d) with the final quality score of the extracted CAT as 96. There are subtle differences in some branches, such as side branches on the pRCA, but these small branches are not important and they are below the maxima for the coarse improvement. These small branches are kept in the extraction. All the removed branches are recovered successfully.

We didn’t perform experiments for the remaining 93 cases, since it is to be expected that similar to the 5 selected cases, the proposed method is able to fully recover a CAT if there are continuous vessel-like structures in the binary vesselness image.


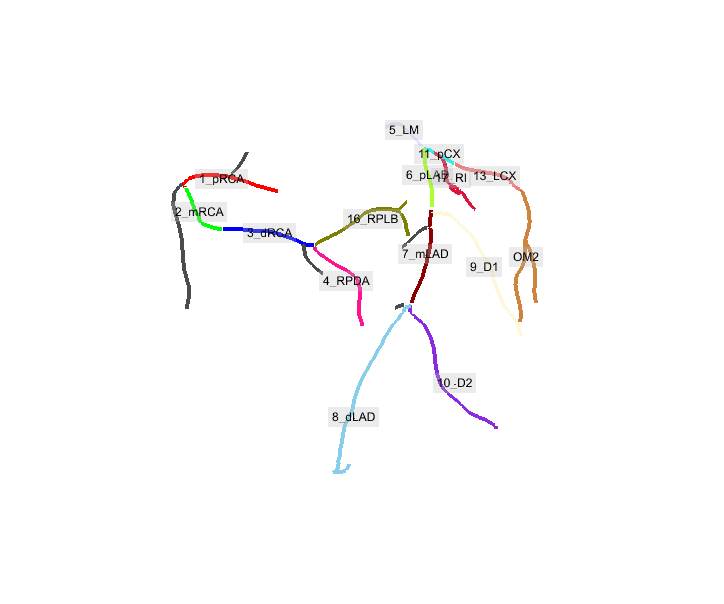

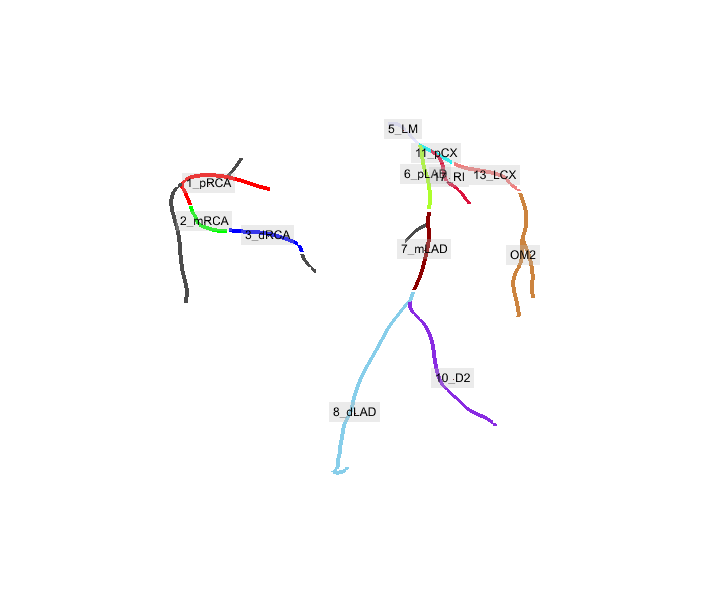


(a). Initial CAT; ***S*=96**

(b). Artificially Pruned CAT; ***S*=85**

(d). Auto-Recovered Final CAT; ***S*=96**


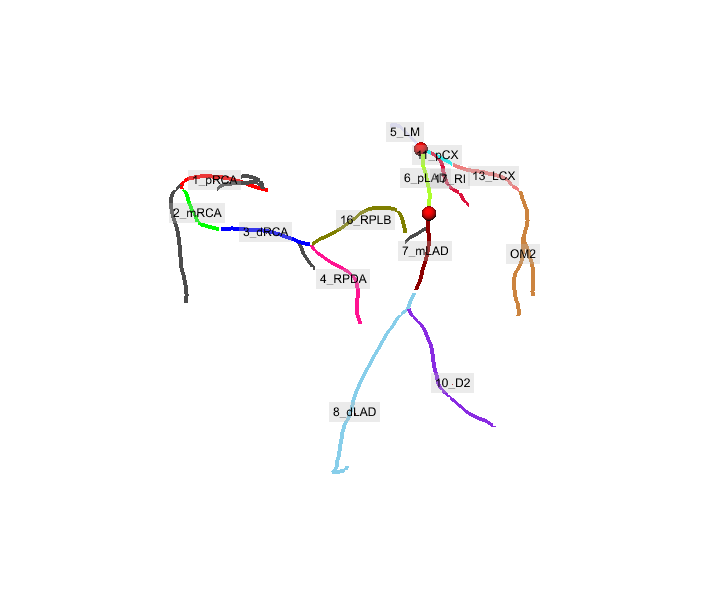

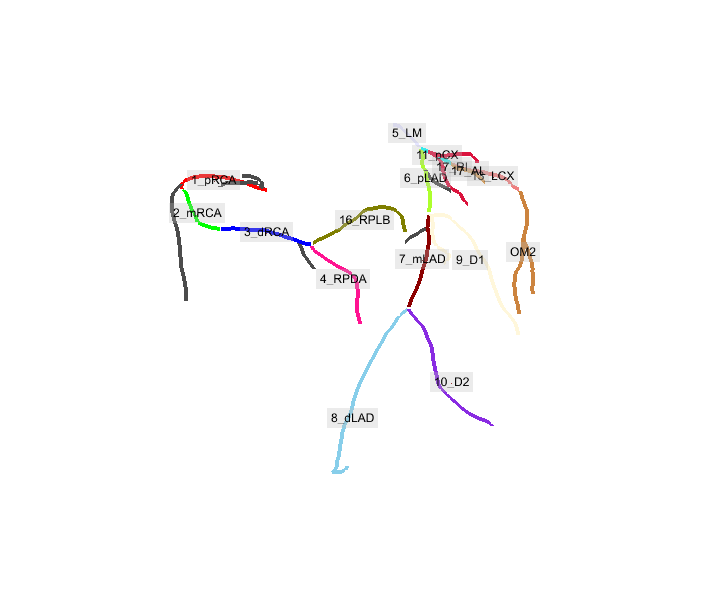


(c). Auto-Recovered CAT; ***S*=91**

1. Automatically recover an artificially pruned CAT to its initial fully extracted CAT. (a) A fully extracted CAT. (b) The artificially pruned CAT by removing the RPDA, RPLB and D1 branch. (c) The automatically improved CAT extending from the RCA to get the RPDA and RPLB. (d) The automatically improved CAT extending from the pLAD to get the D1. *S* is the quality score for each CAT. CAT= Coronary Artery Tree. RPDA= Right Posterior Descending Artery; RPLB= Right Posterior Lateral Branch; RD= Right Dominant; CAT= Coronary Artery Tree; RCA= Right Coronary Artery.
